# The *IFITM5* mutation c.-14C > T results in an elongated transcript expressed in human bone; and causes varying phenotypic severity of osteogenesis imperfecta type V

**DOI:** 10.1186/1471-2474-15-107

**Published:** 2014-03-27

**Authors:** Syndia Lazarus, Aideen M McInerney-Leo, Fiona A McKenzie, Gareth Baynam, Stephanie Broley, Barbra V Cavan, Craig F Munns, Johannes Egbertus Hans Pruijs, David Sillence, Paulien A Terhal, Karena Pryce, Matthew A Brown, Andreas Zankl, Gethin Thomas, Emma L Duncan

**Affiliations:** 1 The University of Queensland Diamantina Institute, Translational Research Institute, Woolloongabba, QLD 4102, Australia; 2 UQ Centre for Clinical Research, University of Queensland, Herston, QLD 4029, Australia; 3 Department of Endocrinology, Royal Brisbane & Women’s Hospital, Herston, QLD 4029, Australia; 4 Genetic Services of Western Australia, Subiaco, WA 6008, Australia; 5 School of Paediatrics and Child Health, The University of Western Australia, Crawley, WA 6009, Australia; 6 Cebu Institute of Medicine, Cebu City, Cebu 6000, Philippines; 7 Bone & Mineral Medicine, Endocrinology, The Children’s Hospital at Westmead, Westmead, NSW 2145, Australia; 8 Connective Tissue Dysplasia Management Service The Sydney Children’s Hospital Network, Westmead, NSW 2145, Australia; 9 Department of Orthopaedics, University Medical Centre Utrecht, 3584 EA Utrecht, The Netherlands; 10 Discipline of Genetic Medicine, Sydney Children’s Hospital Clinical School, University of Sydney, Westmead, NSW 2145, Australia; 11 Department of Medical Genetics, University Medical Centre Utrecht, 3508 Utrecht, The Netherlands

**Keywords:** Osteogenesis imperfecta, Interferon-induced transmembrane protein 5 (IFITM5), Bone-restricted interferon-induced transmembrane protein-like protein (BRIL), Hyperplastic callus, Radial head dislocation

## Abstract

**Background:**

The genetic mutation resulting in osteogenesis imperfecta (OI) type V was recently characterised as a single point mutation (c.-14C > T) in the 5’ untranslated region (UTR) of *IFITM5*, a gene encoding a transmembrane protein with expression restricted to skeletal tissue. This mutation creates an alternative start codon and has been shown in a eukaryotic cell line to result in a longer variant of IFITM5, but its expression has not previously been demonstrated in bone from a patient with OI type V.

**Methods:**

Sanger sequencing of the *IFITM5* 5’ UTR was performed in our cohort of subjects with a clinical diagnosis of OI type V. Clinical data was collated from referring clinicians. RNA was extracted from a bone sample from one patient and Sanger sequenced to determine expression of wild-type and mutant *IFITM5*.

**Results:**

All nine subjects with OI type V were heterozygous for the c.-14C > T *IFITM5* mutation. Clinically, there was heterogeneity in phenotype, particularly in the manifestation of bone fragility amongst subjects. Both wild-type and mutant *IFITM5* mRNA transcripts were present in bone.

**Conclusions:**

The c.-14C > T *IFITM5* mutation does not result in an RNA-null allele but is expressed in bone. Individuals with identical mutations in *IFITM5* have highly variable phenotypic expression, even within the same family.

## Background

Osteogenesis imperfecta (OI) is a heterogeneous group of disorders characterised by fragile bones. An association of OI with hyperplastic callus was first reported by Battle and Shattock in 1908 [1], and other papers over the past century highlighted familial occurrence [1-3]. A further association with calcification/ossification of the interosseous membranes limiting pronation/supination of the forearms was also recognized [2-5]. Further confirmation that this might be a distinct genetic entity was provided by Glorieux *et al.* in 2000 who noted a distinctive histomorphometric pattern in iliac crest bone biopsies [4].

OI type V is an autosomal dominant disorder occurring either de novo or by inheritance from an affected parent. Although its most conspicuous feature is the propensity to form hyperplastic callus, not all patients develop this feature [4-8]. However, when hyperplastic callus does occur, historic reports indicate it may be so exuberant as to lead to major functional complications – in some cases it has resulted in amputation when it has been misdiagnosed as an aggressive malignancy [2].

Through whole exome sequencing, two groups recently identified a single point mutation (c.-14C > T) in the 5’ untranslated region (UTR) of the gene encoding interferon-induced transmembrane protein 5 (IFITM5, also known as Bril) as the cause of OI type V (GenBank ID rs373183215) [9,10]. This mutation creates a novel upstream start codon in-frame with the wild-type *IFITM5* open reading frame, predicted to add five amino acid residues (Met-Ala-Leu-Glu-Pro) to the encoded protein*. In vitro* experiments demonstrated that eukaryotic cells initiated transcription from this alternative start codon [9,10]. However, it is not known whether the mutant allele is transcribed in human bone.

Expression of wild-type *IFITM5* is restricted to bone and cartilage [9,11], hence its alternative name Bone-restricted interferon-induced transmembrane protein-like protein (BRIL) [11]. However, little is known about the mechanism of action of IFITM5 and thus how the mutation contributes to the OI type V phenotype is also unclear.

We investigated our cohort of OI type V subjects for mutations in *IFITM5*. We also examined bone tissue from an individual with OI type V for expression of mutant transcripts, which has not been reported previously.

## Methods

Individuals with OI type V and their families were recruited from Australia and abroad through the Australian Bone Dysplasia Registry at the University of Queensland. Clinical information was provided by the contributing clinicians. All recruited individuals or their legal guardians gave written consent for genetic testing and de-identified information to be published. The study was approved by the University of Queensland Ethics Committee (HREC reference number UQ #2011000876).

Genomic DNA was obtained from all individuals from either blood or saliva by standard techniques, either by a chloroform extraction method or the Oragene Self-collection kit (Genotek, Canada). A bone sample was obtained from patient 176.3 during femoral intramedullary rod replacement surgery. RNA was extracted from bone using TRIzol Reagent (Invitrogen, USA). cDNA was synthesized using the Bioline cDNA Synthesis kit (Bioline, UK). PCR amplification and Sanger sequencing of exon 1 (including the 5’ UTR) was performed using previously described primers [9]. Primer sequences used for cDNA amplification were F 5′-ACCAGTCTGAGTGTGGAAGA-3′ and R 5′-CTGAACACCGACCAGATCAA-3′. Sequencing was performed on the GeneticAnalyzer 3130 (Applied Biosystems, USA) using BigDye v3.1 reagents, and analysed using the manufacturer’s software.

## Results and discussion

Nine cases with OI type V were recruited. Their clinical details are summarised in Table 1. Individuals 185.3 and 185.4 were an affected mother and son, respectively. One further individual (91.3) possibly had an affected parent but this was not definitively confirmed. Additional unaffected family members were recruited for individuals 73.3, 88.3 and 176.3.

**Table 1:** Clinical data of individuals with OI type V

**Individual**	**Age (years)**	**Sex**	**Age at first fracture**	**Number of clinical fractures**	**Age at which elbow deformity noted**	**Age at diagnosis of OI**	**Age at diagnosis of OI type V**	**Forearm interosseous membrane calcification and radial head dislocation**	**Hyperplastic callus**	**Height (Z-score)**	Baseline BMD^*^	**Physical activity**
73.3	10	F	Birth	~18	1 month	Birth	5 years	Present	Present	−2.37	TB −2.69, LS −4.87 at 14 months	Walking unaided
88.3	11	M	Birth	>20	4 years	Birth	4 years	Present	Present	0.359	TB −3 at 10 years	Walking unaided
91.3	25	F	3 years	~8	<1 year	9 years	9 years	Present	Absent	−1.171	TB −2.90, LS −2.73 at 6 years	NA
150.3	61	M	6 weeks	>15	30 years	6 weeks	60 years	Present	Absent	−10.69	NA	NA
176.3	14	F	<12 months	~20	NA	13 months	12 years	Present	Present	−3.94	NA	Wheelchair to mobilise
181.3	7	M	<11 months	20-30	NA	13 months	7 years	Present	Present	−1.07	LS −7.8 at 13 months	Walking for 45 min
182.3	8	F	<1 year	~35	4 years	2 years	8 years	Present	Present	−3.0	LS −4.7 at 2 years	Walking for 30 min intervals
185.3	32	F	Never clinically fractured	0	1 month	31 years	31 years	Present	Absent	−2.79	NA	Walking unaided
185.4	5	M	2 years	3	2.5 years	4 years	4 years	Present	Absent	−1.10	NA	Walking unaided

NA = Not available.

*Baseline BMD prior to bisphosphonate therapy expressed as z-score for total body (TB) and/or lumbar spine (LS) taken at the stated age.

All subjects displayed the characteristic elbow deformity with radial head dislocation - indeed, the diagnosis of OI type V in 185.3 was only made when it was noted that she had the same characteristic elbow deformity as her affected son (185.4) as she had never clinically fractured. In the six cases with a recorded age at which the elbow deformity was first noted, it was present before diagnosis of OI type V, but had been noted before the first fracture in only two of the six subjects. Calcification of the forearm interosseous membrane was evident in all subjects. Five subjects developed hyperplastic callus. In contrast, bone fragility was quite variable, evidenced by the number of clinical fractures reported. For example, individual 182.3, aged eight years, reported approximately 35 clinical fractures; in contrast, individual 185.3, aged 32 years, had never had a clinical fracture. Heterogeneity was also seen in height and physical activity level. None of the subjects had blue sclerae or opalescent dentine.

Sanger sequencing of *IFITM5* showed that all individuals with OI type V were heterozygous for the autosomal dominant c.-14C > T mutation recently identified as the cause of this disease [9,10]. The mutation segregated with phenotype in the three families in whom unaffected members were available.

**Figure 1:**
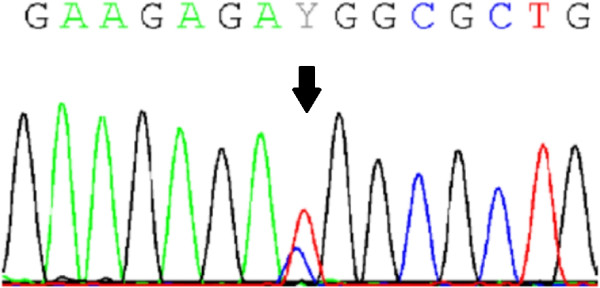
Sanger sequencing of cDNA from bone of individual 176.3, showing expression of both wild-type and mutant transcripts 14 bases upstream of the start codon (arrow).

The bone sample from individual 176.3 was analysed for expression of wild-type and mutant *IFITM5* mRNA. Sanger sequencing of synthesized cDNA confirmed the presence of both wild-type and mutant transcripts in the sample (shown in Figure 1). This is the first demonstration that mutant IFITM5 is expressed in human bone.

Our data supports the current literature that *IFITM5* mutation c.-14C > T is the cause of OI type V. All affected individuals in our cohort were heterozygous for this mutation. The mutation occurred *de novo* in the three simplex cases for whom parental DNA was available. Cho *et al.* and Semler *et al.* demonstrated this mutation in their cohorts of 19 and two individuals with OI type V respectively. Four other groups have subsequently found the same mutation in their entire cohorts [12-15]. Including our cohort, all 95 individuals with OI type V described in the literature who have been investigated for this mutation have been heterozygous for this mutation. Such conservation of a single disease-causing mutation across geographically distinct cohorts is unusual although a similar example in skeletal disease is the 617G > A mutation in the activin A type I receptor gene (ACVR1) in fibrodysplasia ossificans progressive, another disease with abnormal ossification [16].

Whilst not present in all affected individuals reported to date [17], ossification of non-skeletal tissue (in particular, the forearm interosseous membrane) appears to be one of the most characteristic features of this bone disease and was present in all our subjects. An abnormal appearance of the elbow from radial head dislocation may also be an early and characteristic deformity and was also evident in our entire cohort. Published case series report 76-100% of affected individuals have calcification of the interosseous membrane [4,6,8,9,12,13], and 36-88% have radial head dislocation [4,8,9,13]. Conversely, a series of 489 upper limb radiographs from individuals with OI found 86% of those with type V had radial head malalignment compared with 0 to 29% of those with other OI types [18]. In our cohort, five individuals (56%) had a history of hyperplastic callus; previous case series report 8-65% develop this feature [4-6,8,12,13].

Bone fragility appears to be more variable. Most strikingly, one adult patient had no history of clinical fracture. The cohort reported by Cho and colleagues also included individuals with few fractures [9]. Possibly, individuals with a milder OI type V phenotype without a marked fracture history may have been missed, skewing the current understanding of the severity of the disorder. Height [12,13] and bone mineral density (BMD) [12] have also been shown to vary widely; our own data were difficult to interpret in this regard due to bisphosphonate use and varying methods of BMD measurement.

Classification of individuals with OI has become increasingly complex, as the molecular basis of various forms of OI has been elucidated [19-22]. Sequencing of individuals with OI to determine their causative mutation is currently a research rather than clinical tool, but efficient and accurate sequencing may soon become routine clinical activity and facilitate a wider appreciation of the pathogenesis of bone fragility. OI has been generally considered a disorder of collagen or collagen processing [21,22]; where OI type V best fits in this understanding will become clearer as our knowledge of IFITM5 function increases. With respect to this point, it is quite surprising that the phenotypes of individuals identified with the same c.-14C > T mutation vary so significantly in their bone fragility yet so uniformly manifest the elbow deformity and calcification of the forearm interosseous membrane. Presumably other factors interacting with IFITM5 govern the diversity of disease presentation. Phenotypic variability in individuals carrying the same mutation is not in itself is unique to OI type V but has been observed with other forms of OI, possibly due to the effect of modifier genes [23].

We have shown that the mutant form of *IFITM5* is transcribed into mRNA in bone, and this mutation therefore does not result in an RNA-null allele. This has not previously been demonstrated. Unfortunately, accurate quantification of mRNA transcripts could not be performed, as this would require large volumes of bone sample from affected cases. The c.-14C > T mutation has been demonstrated to produce a premature start codon that results in a mutant form of IFITM5 five amino acids longer than the wild-type in *in vitro* studies using HEK293T cells [9,10]. Whilst we have shown the mutant *IFITM5* gene is transcribed in human bone, the longer form of IFITM5 protein has not yet been demonstrated in bone; this will be challenging in human bone due to scarcity of clinical sample.

IFITM5 is a small transmembrane protein, with expression restricted to skeletal tissue, particularly to areas undergoing early ossification [11,24]. In both osteoblast cell lines and primary cultures, *IFITM5* expression is tightly correlated with differentiation and mineralisation with highest expression in the early mineralisation stage [11,24]. *Ifitm5* overexpression in osteoblast cell lines enhanced mineralisation and conversely decreased mineralisation was seen with shRNA knockdown of *Ifitm5*[11]. Thus, IFITM5 appears to be an osteoblast-specific membrane protein involved in early bone mineralisation whose interaction partners are as yet unidentified. It is interesting to speculate that the role of *IFITM5* in mineralisation might contribute significantly to the ectopic ossification and hyperplastic callus seen in individuals with OI type V.

Surprisingly, *Ifitm5*-null mice did not have significantly different morphometric parameters of the tibia compared with heterozygous littermates [24]. Semler *et al.* reported two individuals with genomic deletions resulting in haploinsufficiency of *IFITM5* (in addition to deletion of other genes), both of whom had skeletal manifestations (short stature; microcephaly, short phalanges and clinodactyly) but not the phenotype of OI type V [10]. These studies suggest that the clinical phenotype of OI type V is not due to a loss of function of IFITM5. Our expression data are consistent with this suggestion and support the premise that OI type V is likely to arise from a gain of function of this mutation.

A recent study suggested *IFITM5* expression may be regulated by natural antisense transcripts [25]. Whilst our data show both mutant and wild-type *IFITM5* are expressed in bone, the relative amounts of each transcript could not be accurately quantified. However, as the antisense transcripts identified by Liu *et al.* were complementary to nucleotides distant to the 5’ UTR, it seems unlikely that this mechanism of gene regulation would be affected by the c.-14C > T mutation.

## Conclusions

Our data support the hypothesis that the phenotype of OI type V results specifically from expression of a mutant, longer form of IFITM5 by osteoblasts. However, despite a shared aetiology, bone fragility of affected individuals is highly variable though almost all affected individuals manifest interosseous membrane ossification with resultant elbow deformity. Further work is needed to elucidate the role of IFITM5 in osteoblast differentiation and matrix mineralisation to understand the mechanism by which the c.-14C > T mutation results in OI type V. Furthermore, the elucidation of the mechanism resulting in “explosive” hyperplastic callus formation may lead to a specific pharmacological intervention to prevent progression of this devastating manifestation. In addition, given the transmembrane location of IFITM5 it may be a potential therapeutic target for other bone disorders such as fibrodysplasia ossificans progressiva and other forms of ectopic ossification.

## Abbreviations

BMD: Bone mineral density; BRIL: Bone-restricted interferon-induced transmembrane protein-like protein; IFITM5: Interferon-induced transmembrane protein 5; OI: Osteogenesis imperfecta; UTR: Untranslated region.

## Competing interests

All authors state that they have no conflicts of interest to disclose.

## Authors’ contributions

Sample acquisition: SL, AZ, AMML, FAM, GB, SB, BVC, CFM, JEHP, DS, PAT, MAB, ELD. DNA processing and sequencing: SL, KP, GT, MAB, ELD. Analysis: SL, ELD. Project design: SL, AZ, GT, MAB, ELD. Drafting manuscript: SL, GT, ELD. Revising manuscript content and approving final version of manuscript: SL, AZ, AMML, FAM, GB, SB, BVC, CFM, JEHP, DS, PAT, KP, GT, MAB, ELD. ELD takes responsibility for the integrity of the data analysis. All authors read and approved the final manuscript.

## Pre-publication history

The pre-publication history for this paper can be accessed here:

http://www.biomedcentral.com/1471-2474/15/107/prepub
